# Global traumatic brain injury intracranial pressure: from monitoring to surgical decision

**DOI:** 10.3389/fneur.2024.1423329

**Published:** 2024-09-17

**Authors:** Dan Zhang, Yanzhi Sheng, Chengbin Wang, Wei Chen, Xiaofeng Shi

**Affiliations:** ^1^Longgang Central Hospital of Shenzhen, Guangdong, China; ^2^Shenzhen College of Clinical Medicine, Guangzhou University of Chinese Medicine, Guangdong, China

**Keywords:** traumatic brain injury, intracranial pressure, decompressive craniectomy, TBI management, Glasgow Coma Scale

## Abstract

Traumatic brain injury (TBI) is a significant global public health issue, heavily impacting human health, especially in low-and middle-income areas. Despite numerous guidelines and consensus statements, TBI fatality rates remain high. The pathogenesis of severe TBI is closely linked to rising intracranial pressure (ICP). Elevated intracranial pressure can lead to cerebral herniation, resulting in respiratory and circulatory collapse, and ultimately, death. Managing intracranial pressure (ICP) is crucial in neuro-intensive care. Timely diagnosis and precise treatment of elevated ICP are essential. ICP monitoring provides real-time insights into a patient’s condition, offering invaluable guidance for comprehensive management. ICP monitoring and standardization can effectively reduce secondary nerve damage, lowering morbidity and mortality rates. Accurately assessing and using true ICP values to manage TBI patients still depends on doctors’ clinical experience. This review discusses: (a) Epidemiological disparities of traumatic brain injuries across countries with different income levels worldwide; (b) The significance and function of ICP monitoring; (c) Current status and challenges of ICP monitoring; (d) The impact of decompressive craniectomy on reducing intracranial pressure; and (e) Management of TBI in diverse income countries. We suggest a thorough evaluation of ICP monitoring, head CT findings, and GCS scores before deciding on decompressive craniectomy. Personalized treatment should be emphasized to assess the need for surgical decompression in TBI patients, offering crucial insights for clinical decision-making.

## Introduction

Traumatic brain injury (TBI) is the leading cause of disability and death among young people worldwide. TBI often leads to increased intracranial pressure, reducing cerebral perfusion pressure and causing cerebral ischemia (1). In particular within low-and middle-income countries, the incidence of TBI is the highest, and the mortality rate is also the highest (2). Severe TBI (sTBI) Management depends on mitigating secondary brain injury (3–5). In this scenario, medically refractory intracranial hypertension after sTBI is the most common cause of death (6). Monitoring intracranial pressure (ICP) is essential in managing severe traumatic brain injury, guiding both pharmacological and surgical treatments (7). The National Brain Trauma Foundation guidelines recommend the use of invasive ICP monitoring (IIB level of evidence) (8). To reduce secondary brain damage, high ICP is associated with high mortality and dysfunction in TBI patients. It is currently believed that patients with severe TBI require immediate treatment when the ICP is >22 mmHg (8). However, there has been controversy about this threshold in clinical management. At present, there is no level I evidence to support the use of clinical interventions to target a specific ICP threshold (9). Decompressive craniectomy (DC) stands as a crucial measure in mitigating intracranial pressure among patients, playing a pivotal role in their management (10, 11). Nevertheless, the efficacy of decompressive craniectomy in the treatment of severe head injury has remained a subject of ongoing controversy (12). Craniectomy decompression has the potential to trigger a range of intricate complications, it can even result in severe sequelae (13). Despite the ongoing controversy surrounding it, craniectomy decompression remains the ultimate life-saving procedure for patients suffering from refractory intracranial hypertension. For patients with refractory intracranial hypertension, there is no clear consensus on whether doctors continue to perform conservative treatments for decompression or immediately perform decompressive craniectomy (14). Regarding patients with refractory intracranial hypertension, there exists a lack of a clear consensus among medical professionals on whether to persist with conservative treatment methods for decompression or promptly proceed with decompressive craniectomy (15). The aim of our narrative review is to concisely elaborate on the significance and worth of contemporary intracranial pressure monitoring in patients presenting with elevated intracranial pressure, while simultaneously delving into the prevailing challenges and dilemmas surrounding its application. The main focus of the final review is to summarize the thorough assessment of ICP monitoring, head CT findings, and GCS scores for clinical surgical decisions.

In this review, we conducted a comprehensive evaluation of the disparities in morbidity and mortality rates associated with TBI across various income countries. Furthermore, we delved into the advancements made in ICP monitoring among TBI patients and discussed the application of ICP monitoring across different income nations. At the same time, the role of decompressive craniectomy in patients with TBI is summarized. Finally, we put forward ideas for the direction of future research and clinical services.

## The epidemiology of TBI

**Figure 1:**
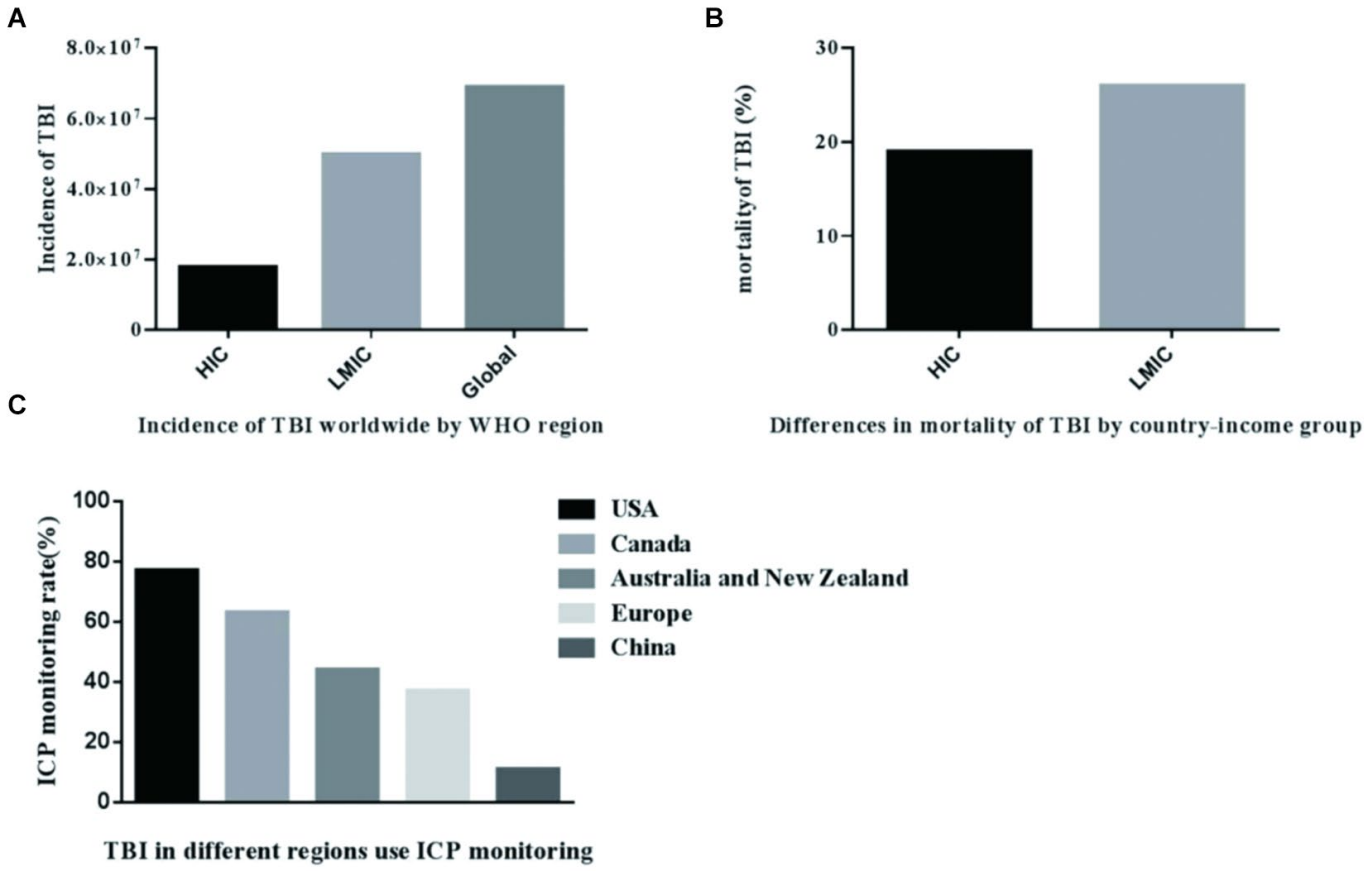
Incidence rate of TBI in countries with different income levels in the world. **(A)** Incidence of TBI worldwide WHO region. **(B)** Differences in mortality of TBI by country-income group. **(C)** TBI in different regions use ICP monitoring.

The majority of the global population resides in low-and middle-income countries (LMIC), where the prevalence of TBI is very high. TBI poses a significant public health challenge, imposing a substantial economic burden on both nations and the world at large (16). The incidence of TBI in LMIC is triple that of high-income countries (HIC), as indicated by available data (17). Utilizing mathematical modeling, researchers have estimated the incidence of TBI in nations with varying income levels globally. The incidence of TBI in low-and middle-income countries accounts for 72% of the global incidence of TBI, and the results are listed in Figure 1A (17). In these countries, acquiring adequate healthcare resources remains a significant challenge. People living in low-and middle-income nations face a much higher risk of fatality from severe TBI, with the likelihood being twice as high as that in high-income countries (18). The total number of deaths is shown in Figure 1B. The decrease in mortality rates among patients with severe TBI in high-income countries may potentially stem from disparities in the quality and accessibility of medical care. TBI patients frequently exhibit a concomitant elevation in ICP. According to estimates, the ICP monitoring rate in the United States is 77.4% (19), Australia and New Zealand is 44.5% (20), Canada is 63% (21), and Europe is 37% (22), 11% in China (23), the data is shown in Figure 1C. In high-income countries, the rate of brain injuries among the elderly is gradually climbing, with a growing proportion of these injuries being caused by falls (24). In low-and middle-income countries, road traffic collisions continue to top the list as the leading cause of brain trauma (25). These epidemiological data consistently demonstrate significant disparities in the occurrence of TBI between high-income countries and their low-and middle-income counterparts. For low-and middle-income countries, individualized treatment may be required based on the clinical experience of clinicians. In comparison to HICs, the scarcity of intensive care capabilities among neurosurgeons in LMICs poses a significant hindrance to effective management, given that LMICs often have limited access to intensive care, and even in capable situations. The available resources are also very limited (26). Consequently, in numerous low-and middle-income countries, patients suffering from severe TBI are unable to receive proactive and comprehensive intensive care. Nursing interventions such as body position and tracheal suctioning can affect the change of intracranial pressure in patients with TBI (27, 28). Nursing operations should be carried out under close monitoring (2). In developed nations replete with a plethora of medical resources, TBI might exhibit relatively consistent trends, irrespective of external factors such as nursing interventions. However, In LMICs, limited medical resources often lead to insufficient numbers and capacity of medical personnel to meet patient needs (29). The implementation of intracranial pressure management methods recommended by guidelines, such as deep analgesia, sedation, and muscle relaxation, is frequently difficult to implement (8). Because analgesia and sedation necessitate continuous vigilant bedside monitoring by nurses, muscle relaxation demands a titrated treatment. The vast majority of patients failed to undergo therapeutic interventions deep sedation for the purpose of reducing intracranial pressure, nor were they administered muscle relaxation titration therapy (30). This led to significant variability in the ICP levels observed among TBI patients. Therefore, TBI management guidelines based on intracranial pressure are difficult to be properly applied in low-and middle-income countries. A multicenter randomized controlled trial (RCT) conducted in the integrated ICU of LMIC (such as Bolivia and Ecuador) compared the differences between two TBI management strategies, one of which was based on ICP and the other did not (31). In view of the increasing differences in resources and epidemiology between LMIC and HIC, the trials conducted in this case may not be enough to show that management based on ICP monitoring has no advantages (32). In the future, conducting more RCT studies for low-TBI populations in low-and middle-income countries is a key direction.

However, a preponderance of studies and clinical trials pertaining to TBI are typically conducted in high-income nations, and some patients recruited to LMIC should be given more attention (19). Translating HIC research findings to LMICs is challenging due to their limited access to intensive care and scarce resources, even when available. A recent survey revealed that LMIC neurosurgeons’ lack of intensive care competence, in contrast to HICs, poses a significant hurdle in patient management (33).

## The importance and role of intracranial pressure monitoring

Neurosurgery patients with TBI are critically patients, and their condition changes rapidly (34). ICP is one of the main complications of traumatic brain injury (33). A substantial body of cohort studies has consistently demonstrated that ICP is independently associated with an elevated risk of mortality and poor prognosis. Therefore, the core of strict management of TBI patients lies in the management of elevated ICP (35–37). According to the changes in ICP, combined with the changes in the patient’s consciousness, pupils and vital signs, it can be timely and accurately judged and found changes and the development of the patient’s condition and timing of effective rescue (38). ICP monitoring can accurately reflect changes in intracranial pressure (39). According to the Fourth Edition Guidelines of the Trauma Foundation (8), TBI patients presenting with a GCS score ranging from 3 to 8 and exhibiting abnormal head CT scans should undergo ICP monitoring upon regaining consciousness. Abnormal CT scans of the head typically encompass a range of pathologies, including hematoma, contusion, swelling, herniation, and compression of the basal cistern (40). Furthermore, for patients who have sustained severe TBI and been admitted to the hospital with normal cranial CT scans, intracranial pressure ought to be meticulously monitored if they exhibit ≥2 of the following characteristics: age over 40 years; unilateral or bilateral limb dyskinesia; systolic blood pressure (blood pressure) < 90 mmHg (41). The consensus among experts, as outlined in these guidelines, underscores the necessity for ICP monitoring and management of patients suffering from TBI. In an observational study, the implementation of ICP monitoring correlated with a diminished risk of fatality (42). A study utilizing a vast prospectively collected database analyzed the impact of ICP reduction interventions on the 2-week mortality of patients with severe TBI treated with or without an ICP monitor. In patients with severe TBI who are treated with intracranial hypertension, the use of an ICP monitoring can significantly reduce the mortality rate compared with patients who are not treated with an ICP monitoring (43). Another study delved into the trials and case series pertaining to the treatment of severe closed TBI, meticulously analyzing both the mortality rates and the favorable outcomes 6 months post-injury. Active ICP monitoring and treatment of patients with severe TBI can significantly improve the prognosis (44). A prospective observational study meticulously assessed the impact of intraventricular ICP monitoring on the prognosis of elderly patients suffering from severe TBI. It has been found through research that the implementation of intraventricular ICP monitoring can mitigate the gravity of in-hospital mortality rates among geriatric patients who have sustained TBI, and improve their 6-month post-injury prognoses (45). A Meta-Analysis since the third edition of “Brain Injury Treatment Guidelines” shows the results (Including “Indications for Intracranial Pressure Monitoring”), with severe TBI monitored by ICP, an excellent survival rate has been observed (46). In cases of severe TBI, an elevation in ICP is intricately linked to mortality and an unfavorable clinical prognosis (47). The main goal of ICP monitoring in TBI is to be able to detect secondary injuries early and implement therapeutic interventions immediately (48). Although a randomized study showed that continuous monitoring of ICP in patients with TBI did not improve the prognosis compared with nursing care, however, based on imaging and clinical examination (49), there are significant differences in treatment plans between the two groups in this study. It does not endeavor to evaluate the inherent worth of ICP monitoring, but rather employs two distinct methodologies to ascertain the effectiveness of intracranial hypertension management. The core value of ICP monitoring is to guide the correct treatment strategy, so there is no need to use the evidence in this study to show that ICP monitoring has no value or can be used as an argument against the use of ICP monitoring (12). The implementation of long-term continuous ICP monitoring is advantageous, as the patterns of ICP variation can serve as a guiding principle for individualized treatment (50). In addition, ICP monitoring combined with other nervous system monitoring helps to understand the pathophysiological process of injury (51). Drawing upon clinical practice and literature reports, we provide a overview of the specific performance of ICP monitoring. The value of the following aspects: (a) Early judgment of the patient’s prognosis; (b) Judge whether to perform craniotomy and decompression, and determine the surgical strategy; (c) Early diagnosis of delayed intracranial hematoma, as an early warning; (d) Guide clinical treatment, such as the application of dehydration drugs, the time course of mild hypothermia treatment; and (e) Determine the surgical effect of bone flap surgery. A series of typical clinical studies are shown in Table 1, which shows the application value of ICP monitoring in traumatic brain injury.

**Table 1:** Clinical trial of useful value of ICP monitoring in patients with traumatic brain injury.

	Sample	Number of patients	Study design	Outcomes
Chiara Robba et al. (2021) (85)	Patients aged 18 years or older who were admitted to the ICU with either acute brain injury due to primary haemorrhagic stroke or traumatic brain injury	2,395	Prospective observational cohort study	6 month mortality was lower in patients who had ICP monitoring (441/1318 [34%]) than in those who were not monitored (517/1049 [49%]; *p* < 0·0001).
Lele A et al. (2019) (86)	Patients over 18 year with severe TBI (admission Glasgow coma scale score < 8) who received tracheal intubation for at-least 48 h were examined	200	a secondary analysis of a prospective cohort study	ICP monitor placement without cerebrospinal fluid drainage within 72 h of admission was associated with reduced in-patient mortality.
Qiang Yuan et al. (2016) (87)	Patients with severe diffuse TBI (GCS score on admission <9 and Marshall Class II-IV)	482	Retrospective cohort	ICP monitor placement was associated with a significant decrease in 6-month mortality after adjustment for the baseline risk profile and the monitoring propensity of patients with diffuse severe TBI, especially those with GCS scores of 3 to 5 or of Marshall computed tomography classification IV.
Qiang Yuan et al. (2015) (88)	Moderate or severe traumatic brain injury patients who were more than 14 years old	1,443	Retrospective observational multicenter study.	ICP monitoring was significantly associated with an improved 6-month mortality for patients with TBI who had a GCS score of 3–5 at admission, had a GCS score of 9–12 at admission that dropped to 3–8 within 24 h after injury
Arash Farahvar et al. (2012) (43)	Patients with severe TBI (GCS Score < 9) treated for intracranial hypertension	1,446	Prospectively cohort	In patients with severe TBI treated for intracranial hypertension, the use of an ICP monitor is associated with significantly lower mortality when compared with patients treated without an ICP monitor
Bennett TD et al. (2012) (89)	Children (age < 18 years) with TBI and head/neck Abbreviated Injury Scale (AIS) score ≥ 3 who were ventilated for ≥96 consecutive hours or died in the first 4 days after admission	4,667	Retrospective cohort study	Hospitals that monitor ICP more often and hospitals with higher patient volumes had better patient outcomes
Walter Mauritz et al. (2008) (90)	Patients with severe TBI (GCS < 9)	1856	Prospective multicenter cohort study	The lowest mortality rates (raw and risk-adjusted) were found in the subgroup with the highest rate (91.1%) of ICP monitoring
Lane PL et al. (2000) (91)	Registered cases of TBI	9,001	Study of case records	The insertion of an ICP monitor is associated with a statistically significant decrease in death rate among patients with severe TBI

## The current state of ICP monitoring technology and the future direction

The conventional ICP monitoring technique involves the direct implantation of a pressure sensor within the brain, subsequently connecting the pressure monitoring device to the exterior. This approach boasts the benefits of straightforward operation and precise pressure measurement (52). In contemporary medical practice, two prominent technologies, namely External Ventricular Drains (EVD) and Intraparenchymal Fiberoptic Monitors (IPM), stand as the preferred and recommended technology for intracranial pressure monitoring (53). At the same time, EVD can also drain cerebrospinal fluid to achieve the therapeutic purpose of reducing intracranial pressure (54). EVD is considered the gold standard. The conventional techniques employed for the monitoring of ICP are invasive in nature, which can potentially result in a series of complication. These include, but are not limited to, intracranial infections, intracranial hematomas, and damage to brain tissues (55–58). In addition, the utilization rate of ICP monitoring technology in low-and middle-income nations remains exceptionally low. This may be attributed to the technical intricacies of ICP monitoring, as well as its prohibitively high cost (59). The optimal ICP monitoring device should exhibit precision, dependability, and cost-effectiveness, while concurrently imposing minimal morbidity (60). Therefore, the establishment of a non-invasive and simple bedside ICP monitoring method is of great value for trauma first aid. Prior research has revealed a positive correlation between changes in the optic nerve sheath diameter (ONSD) and ICP (61). However, it is worth noting that measurements of ONSD may encounter interference from the presence of papilledema (62). The Erasmus Medical Center in the Netherlands conducted clinical trials aimed at dynamically tracking alterations in ICP and analyzing whether such changes directly influence the measured value of ONSD. They discovered that the utilization of ultrasound measurement for ONSD in individuals suffering from TBI serves as a swift, straightforward, and efficacious approach (63). Accurate ICP non-invasive monitoring method, with ONSD≥5.0 mm as the critical value, accurately judge the increase of ICP (>20 mmHg) (64). This technology can be used in injured sites, emergency rooms and other places where invasive ICP monitoring cannot be implemented. In addition, compared with other literature reports using CT to measure the diameter of the optic nerve sheath, the ultrasonic measurement method can reflect ICP more simply and in real time, and is practical for clinical use (65). An observational study confirmed that the combination of ONSD and estimated ICP (eICP) using transcranial Doppler may improve the accuracy of estimating the occurrence of intracranial hypertension (66).

In fact, non-invasive ICP monitoring encompasses a diverse array of categories, which are outlined as follows: (1) imaging magnetic resonance, tomography, ONSD and other technologies; (2) indirect transmission of ICP, such as TCD and eyeball and ophthalmic artery methods; (3) monitoring metabolic changes, Such as near-infrared spectroscopy; and (4) the registration of functional activities, such as EEG, visual evoked potentials and auditory potentials (67). Theoretically speaking, the concept of a non-invasive technique for measuring intracranial pressure holds significant appeal, primarily due to its reduced tedious action and can avoid circumvent complications like hemorrhage and infection (68). The utmost priority thing is that the price is not too high. In the future, we aspire to conduct randomized clinical trials to substantiate the clinical impact of utilizing non-invasive techniques for intracranial pressure measurement.

## How to manage the elevated intracranial pressure

### The role of decompressive craniectomy in patients with TBI

Decompressive craniectomy (DC) is a neurosurgical intervention in which a part of the skull is removed to mitigate the pathological escalation of ICP, avert the occurrence of cerebral herniation, and prevent the onset of cerebral tissue ischemia (69). This surgery procedure holds the potential to improve cerebral hemodynamics and oxygenation in patients suffering from elevated ICP (70, 71). Furthermore, in certain instances, it can contribute to the reduction of mortality rates and disabilities among those afflicted with TBI (56). In patients with worsening cerebral edema, it effectively provides extra space for the swollen brain and reduces the risk of further elevation of ICP and brain herniation (57). The basic principle of DC is to remove a part of the skull, transforming the originally closed cranial cavity into a relatively open state, thereby changing the fixed capacity and limited ICP of the cranial cavity (72). Ever since the early 2000s, there has been a concerted effort not only to improve the pre-hospital care for TBI patients but also introduce well-designed programs and implement ICP monitoring. These advancements have collectively transformed the therapeutic environment of DC. The basic principle of decompression surgery is based on the Monro-Kellie law (59). According to this theory, the intracranial volume should be kept constant, and the volume should be compensated by the transfer of cerebrospinal fluid, cerebral blood volume or brain herniation. Removing a variable amount of bone, whether it involves enhancing the openness of the dura mater or augmenting the number through duraplasty, serves as a rapid and efficient approach to expand intracranial volume, thereby effectively reducing intracranial pressure and enhancing intracranial space (73). In recent years, the significant difference in the use of DCs globally has been the practice gap between low - and middle-income countries and high-income countries, which constitutes an important background for clinical decision-making (74). The application of DC surgery in high-income countries is supported by high-quality randomized controlled trials (RCTs) evidence. For example, the study on Neurosurgery at the University of Manitoba in Canada (75) integrated multiple clinical trial results, providing updated guidelines for the application of DCs in sTBI. These studies indicate that timely DC surgery can significantly reduce mortality in patients with refractory intracranial hypertension. High income countries rely on advanced medical technology and comprehensive postoperative management to ensure the safety and effectiveness of DC surgery (76). In contrast, low-income countries face many challenges in the application of DC surgery due to limited medical resources. These countries often lack advanced intracranial pressure monitoring equipment and appropriate nursing techniques, which limits the application of DC surgery (77). However, in emergency situations such as acute traumatic brain injury, DC surgery is still considered an important means of saving lives (78). However, inadequate postoperative management and rehabilitation support may affect the long-term prognosis of patients. Therefore, when formulating a global strategy for DC surgery, it is necessary to fully consider the economic, medical, and social conditions of different countries.

### TBI management: optimal strategy for ICP monitoring

Determining the optimal timing for DC necessitates clinicians to possess a more comprehensive understanding of the ICP situation. This requires a complete ICP management plan. Given the significant individual differences among patients suffering from craniocerebral trauma, relying solely on a singular ICP indicator is evidently inadequate as a means to accurately assess prognosis.

Comprehensively, the value of ICP monitoring lies in real-time reflection of dynamic changes in intracranial pressure, providing key information for clinical diagnosis and treatment decision-making, and improving patient prognosis (79). A recent study has revealed that the score derived from head CT scans and the volume of brain contusion exhibit a correlation with the elevation of ICP (80). Consequently, these influencing characteristics can be harnessed to anticipate the risk of increased ICP in patients with sTBI and corresponding treatment (66). The combination of clinical and head CT examination results that can be used to make management decisions may be an effective strategy, which is also one of the directions of future clinical research. The Glasgow Coma Scale (GCS) provides a convenient method for quickly assessing the level of consciousness of TBI patients (67), but the GCS score alone cannot provide enough information to guide surgical decision-making. Therefore, elucidating the surgical requisites of patients should be combined with this information. Hence, we stress the importance of a comprehensive evaluation of ICP monitoring, head CT findings, and GCS scores for surgical decision-making to ensure a more reliable approach, particularly in specific scenarios. However, in high-income countries, despite the relatively abundant technological resources, the limited availability of technological resources and the significant differences in decision-making between high-tech medical services still exist (81). ICP monitoring, as the “gold standard” that directly reflects changes in intracranial pressure, is limited in its widespread application in all cases due to its high cost and operational complexity (82). In contrast, CT scanning has become the preferred method for preliminary assessment of traumatic brain injury due to its wide availability and relatively low cost (83). However, CT scanning has limitations in dynamically monitoring changes in intracranial pressure, and continuous observation of GCS scores has become an important supplementary tool to help doctors assess patients’ consciousness status and degree of neurological damage (84). Therefore, in the decision-making process, it is necessary to comprehensively consider the patient’s specific condition, treatment goals, resource cost-effectiveness, and potential risks in order to make the optimal treatment choice.

## Conclusion and future directions

ICP monitoring combined with clinical manifestations and head CT imaging results can comprehensively evaluate the patient’s condition. We emphasize the importance of comprehensive evaluation of ICP monitoring, head CT results, and GCS scores for decision-making on whether to perform surgery, providing guidance to clinical doctors based on specific indications to ensure personalized and optimized treatment.

## References

[1] Maegele M. The long journey towards uniform epidemiological monitoring of TBI around the globe. Lancet Neurol. (2019) 18:228–9. doi: 10.1016/S1474-4422(19)30019-5, PMID: 30784547

[2] Kesinger MR Nagy LR Sequeira DJ Charry JD Puyana JC Rubiano AM. A standardized trauma care protocol decreased in-hospital mortality of patients with severe traumatic brain injury at a teaching hospital in a middle-income country. Injury. (2014) 45:1350–4. doi: 10.1016/j.injury.2014.04.037, PMID: 24861416

[3] van Dijck JT Reith FC van Erp IA van Essen TA Maas AI Peul WC. Decision making in very severe traumatic brain injury (Glasgow coma scale 3-5): a literature review of acute neurosurgical management. J Neurosurg Sci. (2018) 62:153–77. doi: 10.23736/S0390-5616.17.04255-2 29125266

[4] Marehbian J Muehlschlegel S Edlow BL Hinson HE Hwang DY. Medical management of the severe traumatic brain injury patient. Neurocrit Care. (2017) 27:430–46. doi: 10.1007/s12028-017-0408-5, PMID: 28573388 PMC5700862

[5] Liu S Wan X Wang S Huang L Zhu M Zhang S. Posttraumatic cerebral infarction in severe traumatic brain injury: characteristics, risk factors and potential mechanisms. Acta Neurochir. (2015) 157:1697–704. doi: 10.1007/s00701-015-2559-5 26306582

[6] Abraham P Rennert RC Gabel BC Sack JA Karanjia N Warnke P. ICP management in patients suffering from traumatic brain injury: a systematic review of randomized controlled trials. Acta Neurochir. (2017) 159:2279–87. doi: 10.1007/s00701-017-3363-1, PMID: 29058090

[7] Rakhit S Nordness MF Lombardo SR Cook M Smith L Patel MB. Management and challenges of severe traumatic brain injury. Semin Respir Crit Care Med. (2021) 42:127–44. doi: 10.1055/s-0040-1716493, PMID: 32916746

[8] Carney N Totten AM O’Reilly C Ullman JS Hawryluk GW Bell MJ. Guidelines for the management of severe traumatic brain injury, fourth edition. Neurosurgery. (2017) 80:6–15. doi: 10.1227/NEU.0000000000001432 27654000

[9] Caplan HW Cox CS. Resuscitation strategies for traumatic brain injury. Curr Surg Rep. (2019) 7:14. doi: 10.1007/s40137-019-0237-x 31205819 PMC6568265

[10] Shutter LA Timmons SD. Intracranial pressure rescued by decompressive surgery after traumatic brain injury. N Engl J Med. (2016) 375:1183–4. doi: 10.1056/NEJMe1609722, PMID: 27604048

[11] Lilja-Cyron A Andresen M Kelsen J Andreasen TH Fugleholm K Juhler M. Long-term effect of decompressive craniectomy on intracranial pressure and possible implications for intracranial fluid movements. Neurosurgery. (2020) 86:231–40. doi: 10.1093/neuros/nyz049, PMID: 30768137

[12] Romner B Grande PO. Traumatic brain injury: intracranial pressure monitoring in traumatic brain injury. Nat Rev Neurol. (2013) 9:185–6. doi: 10.1038/nrneurol.2013.37 23478466

[13] Elwatidy S. Bifrontal decompressive craniectomy is a life-saving procedure for patients with nontraumatic refractory brain edema. Br J Neurosurg. (2009) 23:56–62. doi: 10.1080/02688690802571094, PMID: 19234910

[14] Sahuquillo J Dennis JA. Decompressive craniectomy for the treatment of high intracranial pressure in closed traumatic brain injury. Cochrane Database Syst Rev. (2019) 2019:CD003983. doi: 10.1002/14651858.CD003983.pub3 PMC6953357 31887790

[15] Rusnak M. Traumatic brain injury: giving voice to a silent epidemic. Nat Rev Neurol. (2013) 9:186–7. doi: 10.1038/nrneurol.2013.38 23478463

[16] Yue JK Rick JW Deng H Feldman MJ Winkler EA. Efficacy of decompressive craniectomy in the management of intracranial pressure in severe traumatic brain injury. J Neurosurg Sci. (2019) 63:425–40. doi: 10.23736/S0390-5616.17.04133-9, PMID: 29115100

[17] Dewan MC Rattani A Gupta S Baticulon RE Hung YC Punchak M. Estimating the global incidence of traumatic brain injury. J Neurosurg. (2018) 130:1080–97. doi: 10.3171/2017.10.JNS17352 29701556

[18] De Silva MJ Roberts I Perel P Edwards P Kenward MG Fernandes J. Patient outcome after traumatic brain injury in high-, middle-and low-income countries: analysis of data on 8927 patients in 46 countries. Int J Epidemiol. (2009) 38:452–8. doi: 10.1093/ije/dyn189 18782898

[19] Hesdorffer DC Ghajar J. Marked improvement in adherence to traumatic brain injury guidelines in United States trauma centers. J Trauma. (2007) 63:841-7; discussion 847-8. doi: 10.1097/TA.0b013e318123fc21 PMID: 18090015

[20] Myburgh JA Cooper DJ Finfer SR Venkatesh B Jones D Higgins A. Epidemiology and 12-month outcomes from traumatic brain injury in Australia and New Zealand. J Trauma. (2008) 64:854–62. doi: 10.1097/TA.0b013e3180340e77, PMID: 18404048

[21] Sahjpaul R Girotti M. Intracranial pressure monitoring in severe traumatic brain injury – results of a Canadian survey. Can J Neurol Sci. (2000) 27:143–7. doi: 10.1017/S0317167100052252, PMID: 10830348

[22] Stocchetti N Penny KI Dearden M Braakman R Cohadon F Iannotti F. Intensive care management of head-injured patients in Europe: a survey from the European brain injury consortium. Intensive Care Med. (2001) 27:400–6. doi: 10.1007/s001340000825, PMID: 11396285

[23] Gao G Wu X Feng J Hui J Mao Q Lecky F. Clinical characteristics and outcomes in patients with traumatic brain injury in China: a prospective, multicentre, longitudinal, observational study. Lancet Neurol. (2020) 19:670–7. doi: 10.1016/S1474-4422(20)30182-4 32702336

[24] Peterson AB Kegler SR. Deaths from fall-related traumatic brain injury-United States, 2008-2017. MMWR Morb Mortal Wkly Rep. (2020) 69:225–30. doi: 10.15585/mmwr.mm6909a2, PMID: 32134910 PMC7367089

[25] Bandyopadhyay S Kawka M Marks K Richards GC Taylor EH Sravanam S. Traumatic brain injury-related pediatric mortality and morbidity in low-and middle-income countries: a systematic review. World Neurosurg. (2021) 153:109–130.e23. doi: 10.1016/j.wneu.2021.06.077, PMID: 34166832

[26] Rubiano AM Puyana JC Mock CN Bullock MR Adelson PD. Strengthening neurotrauma care systems in low and middle income countries. Brain Inj. (2013) 27:262–72. doi: 10.3109/02699052.2012.750742, PMID: 23438347

[27] Fan JY. Effect of backrest position on intracranial pressure and cerebral perfusion pressure in individuals with brain injury: a systematic review. J Neurosci Nurs. (2004) 36:278–88. doi: 10.1097/01376517-200410000-00007, PMID: 15524246

[28] Harrois A Anstey JR Deane AM Craig S Udy AA McNamara R. Effects of routine position changes and tracheal suctioning on intracranial pressure in traumatic brain injury patients. J Neurotrauma. (2020) 37:2227–33. doi: 10.1089/neu.2019.6873 32403976

[29] Maas A Menon DK Manley GT Abrams M Akerlund C Andelic N. Traumatic brain injury: progress and challenges in prevention, clinical care, and research. Lancet Neurol. (2022) 21:1004–60. doi: 10.1016/S1474-4422(22)00309-X, PMID: 36183712 PMC10427240

[30] Oddo M Crippa IA Mehta S Menon D Payen JF Taccone FS. Optimizing sedation in patients with acute brain injury. Crit Care. (2016) 20:128. doi: 10.1186/s13054-016-1294-5, PMID: 27145814 PMC4857238

[31] Lazaridis C Foreman B. Management strategies based on multi-modality neuromonitoring in severe traumatic brain injury. Neurotherapeutics. (2023) 20:1457–71. doi: 10.1007/s13311-023-01411-2, PMID: 37491682 PMC10684466

[32] Zacchetti L Magnoni S Di Corte F Zanier ER Stocchetti N. Accuracy of intracranial pressure monitoring: systematic review and meta-analysis. Crit Care. (2015) 19:420. doi: 10.1186/s13054-015-1137-9, PMID: 26627204 PMC4667503

[33] Rubiano AM Carney N Chesnut R Puyana JC. Global neurotrauma research challenges and opportunities. Nature. (2015) 527:S193–7. doi: 10.1038/nature16035, PMID: 26580327

[34] Stocchetti N Maas AI. Traumatic intracranial hypertension. N Engl J Med. (2014) 370:2121–30. doi: 10.1056/NEJMra1208708 24869722

[35] Jahns FP Miroz JP Messerer M Daniel RT Taccone FS Eckert P. Quantitative pupillometry for the monitoring of intracranial hypertension in patients with severe traumatic brain injury. Crit Care. (2019) 23:155. doi: 10.1186/s13054-019-2436-3, PMID: 31046817 PMC6498599

[36] Addis A Baggiani M Citerio G. Intracranial pressure monitoring and management in aneurysmal subarachnoid hemorrhage. Neurocrit Care. (2023) 39:59–69. doi: 10.1007/s12028-023-01752-y, PMID: 37280411 PMC10499755

[37] Baggiani M Graziano F Rebora P Robba C Guglielmi A Galimberti S. Intracranial pressure monitoring practice, treatment, and effect on outcome in aneurysmal subarachnoid hemorrhage. Neurocrit Care. (2023) 38:741–51. doi: 10.1007/s12028-022-01651-8, PMID: 36471182

[38] Shen H Liu H He J Wei L Wang S. Risk factors of prognosis in older patients with severe brain injury after surgical intervention. Eur J Med Res. (2023) 28:479. doi: 10.1186/s40001-023-01473-0, PMID: 37925438 PMC10625240

[39] Le Roux P. Physiological monitoring of the severe traumatic brain injury patient in the intensive care unit. Curr Neurol Neurosci Rep. (2013) 13:331. doi: 10.1007/s11910-012-0331-2 23328942

[40] Currie S Saleem N Straiton JA Macmullen-Price J Warren DJ Craven IJ. Imaging assessment of traumatic brain injury. Postgrad Med J. (2016) 92:41–50. doi: 10.1136/postgradmedj-2014-133211 26621823

[41] Hawryluk G Citerio G Hutchinson P Kolias A Meyfroidt G Robba C. Intracranial pressure: current perspectives on physiology and monitoring. Intensive Care Med. (2022) 48:1471–81. doi: 10.1007/s00134-022-06786-y, PMID: 35816237

[42] Alali AS Fowler RA Mainprize TG Scales DC Kiss A de Mestral C. Intracranial pressure monitoring in severe traumatic brain injury: results from the American College of Surgeons trauma quality improvement program. J Neurotrauma. (2013) 30:1737–46. doi: 10.1089/neu.2012.2802, PMID: 23731257 PMC3796332

[43] Farahvar A Gerber LM Chiu YL Carney N Hartl R Ghajar J. Increased mortality in patients with severe traumatic brain injury treated without intracranial pressure monitoring. J Neurosurg. (2012) 117:729–34. doi: 10.3171/2012.7.JNS111816, PMID: 22900846

[44] Stein SC Georgoff P Meghan S Mirza KL El FO. Relationship of aggressive monitoring and treatment to improved outcomes in severe traumatic brain injury. J Neurosurg. (2010) 112:1105–12. doi: 10.3171/2009.8.JNS09738, PMID: 19747054

[45] You W Feng J Tang Q Cao J Wang L Lei J. Intraventricular intracranial pressure monitoring improves the outcome of older adults with severe traumatic brain injury: an observational, prospective study. BMC Anesthesiol. (2016) 16:35. doi: 10.1186/s12871-016-0199-9 27401211 PMC4940906

[46] Shen L Wang Z Su Z Qiu S Xu J Zhou Y. Effects of intracranial pressure monitoring on mortality in patients with severe traumatic brain injury: a meta-analysis. PLoS One. (2016) 11:e0168901. doi: 10.1371/journal.pone.0168901, PMID: 28030638 PMC5193438

[47] Shen Y Wen D Liang Z Wan L Jiang Q He H. Brain tissue oxygen partial pressure monitoring and prognosis of patients with traumatic brain injury: a meta-analysis. Neurosurg Rev. (2024) 47:222. doi: 10.1007/s10143-024-02439-4, PMID: 38758384 PMC11101534

[48] Le Roux P. Intracranial pressure monitoring and management. Taylor&Francis Group (2016). 26583172

[49] Chesnut RM Temkin N Carney N Dikmen S Rondina C Videtta W. A trial of intracranial-pressure monitoring in traumatic brain injury. N Engl J Med. (2012) 367:2471–81. doi: 10.1056/NEJMoa1207363, PMID: 23234472 PMC3565432

[50] Brasil S Godoy DA Videtta W Rubiano AM Solla D Taccone FS. A comprehensive perspective on intracranial pressure monitoring and individualized management in neurocritical care: results of a survey with global experts. Neurocrit Care. (2024). doi: 10.1007/s12028-024-02008-z, [Online ahead of print]. PMC11599332 38811514

[51] Timofeev I Gupta A. Monitoring of head injured patients. Curr Opin Anaesthesiol. (2005) 18:477–83. doi: 10.1097/01.aco.0000183107.19673.ed 16534279

[52] Hawthorne C Piper I. Monitoring of intracranial pressure in patients with traumatic brain injury. Front Neurol. (2014) 5:121. doi: 10.3389/fneur.2014.00121 25076934 PMC4100218

[53] Stocchetti N Picetti E Berardino M Buki A Chesnut RM Fountas KN. Clinical applications of intracranial pressure monitoring in traumatic brain injury: report of the Milan consensus conference. Acta Neurochir. (2014) 156:1615–22. doi: 10.1007/s00701-014-2127-4, PMID: 24849391

[54] North B Reilly P. Comparison among three methods of intracranial pressure recording. Neurosurgery. (1986) 18:730–2. doi: 10.1227/00006123-198606000-00009 3736801

[55] Guyot LL Dowling C Diaz FG Michael DB. Cerebral monitoring devices: analysis of complications. Acta Neurochir Suppl. (1998) 71:47–9. PMID: 9779141 10.1007/978-3-7091-6475-4_15

[56] Hoefnagel D Dammers R Ter Laak-Poort MP Avezaat CJ. Risk factors for infections related to external ventricular drainage. Acta Neurochir. (2008) 150:209-14; discussion 214. doi: 10.1007/s00701-007-1458-9 18278575

[57] Cnossen MC Huijben JA van der Jagt M Volovici V van Essen T Polinder S. Variation in monitoring and treatment policies for intracranial hypertension in traumatic brain injury: a survey in 66 neurotrauma centers participating in the CENTER-TBI study. Crit Care. (2017) 21:233. doi: 10.1186/s13054-017-1816-9, PMID: 28874206 PMC5586023

[58] Biersteker HA Andriessen TM Horn J Franschman G van der Naalt J Hoedemaekers CW. Factors influencing intracranial pressure monitoring guideline compliance and outcome after severe traumatic brain injury. Crit Care Med. (2012) 40:1914–22. doi: 10.1097/CCM.0b013e3182474bde, PMID: 22488001

[59] Joseph M. Intracranial pressure monitoring in a resource-constrained environment: a technical note. Neurol India. (2003) 51:333–5. https://journals.lww.com/neur/fulltext/2003/51030/intracranial_pressure_monitoring_in_a.5.aspx PMID: 14652432

[60] Schizodimos T Soulountsi V Iasonidou C Kapravelos N. An overview of management of intracranial hypertension in the intensive care unit. J Anesth. (2020) 34:741–57. doi: 10.1007/s00540-020-02795-7, PMID: 32440802 PMC7241587

[61] Montorfano L Yu Q Bordes SJ Sivanushanthan S Rosenthal RJ Montorfano M. Mean value of B-mode optic nerve sheath diameter as an indicator of increased intracranial pressure: a systematic review and meta-analysis. Ultrasound J. (2021) 13:35. doi: 10.1186/s13089-021-00235-5, PMID: 34215966 PMC8253877

[62] Lochner P Czosnyka M Naldi A Lyros E Pelosi P Mathur S. Optic nerve sheath diameter: present and future perspectives for neurologists and critical care physicians. Neurol Sci. (2019) 40:2447–57. doi: 10.1007/s10072-019-04015-x, PMID: 31367861

[63] Trocha G Bonilla A Romero C Palacios J Molano-Gonzalez N. Ultrasound measurement of optic nerve sheath diameter in a healthy adult Colombian population. BMC Neurol. (2023) 23:16. doi: 10.1186/s12883-023-03062-4, PMID: 36639617 PMC9837461

[64] Maissan IM Dirven PJ Haitsma IK Hoeks SE Gommers D Stolker RJ. Ultrasonographic measured optic nerve sheath diameter as an accurate and quick monitor for changes in intracranial pressure. J Neurosurg. (2015) 123:743–7. doi: 10.3171/2014.10.JNS141197, PMID: 25955869

[65] Chen LM Wang LJ Hu Y Jiang XH Wang YZ Xing YQ. Ultrasonic measurement of optic nerve sheath diameter: a non-invasive surrogate approach for dynamic, real-time evaluation of intracranial pressure. Br J Ophthalmol. (2019) 103:437–41. doi: 10.1136/bjophthalmol-2018-312934, PMID: 30361274 PMC6691934

[66] Robba C Pozzebon S Moro B Vincent JL Creteur J Taccone FS. Multimodal non-invasive assessment of intracranial hypertension: an observational study. Crit Care. (2020) 24:379. doi: 10.1186/s13054-020-03105-z, PMID: 32591024 PMC7318399

[67] Robba C Bacigaluppi S Cardim D Donnelly J Bertuccio A Czosnyka M. Non-invasive assessment of intracranial pressure. Acta Neurol Scand. (2016) 134:4–21. doi: 10.1111/ane.12527 26515159

[68] Canac N Jalaleddini K Thorpe SG Thibeault CM Hamilton RB. Review: pathophysiology of intracranial hypertension and noninvasive intracranial pressure monitoring. Fluids Barriers CNS. (2020) 17:40. doi: 10.1186/s12987-020-00201-8, PMID: 32576216 PMC7310456

[69] Khellaf A Khan DZ Helmy A. Recent advances in traumatic brain injury. J Neurol. (2019) 266:2878–89. doi: 10.1007/s00415-019-09541-4, PMID: 31563989 PMC6803592

[70] Fotakopoulos G Gatos C Georgakopoulou VE Lempesis IG Spandidos DA Trakas N. Role of decompressive craniectomy in the management of acute ischemic stroke (review). Biomed Rep. (2024) 20:33. doi: 10.3892/br.2024.1721, PMID: 38273901 PMC10809310

[71] Rossini Z Nicolosi F Kolias AG Hutchinson PJ De Sanctis P Servadei F. The history of decompressive craniectomy in traumatic brain injury. Front Neurol. (2019) 10:458. doi: 10.3389/fneur.2019.00458, PMID: 31133965 PMC6517544

[72] Hutchinson PJ Kolias AG Timofeev IS Corteen EA Czosnyka M Timothy J. Trial of decompressive craniectomy for traumatic intracranial hypertension. N Engl J Med. (2016) 375:1119–30. doi: 10.1056/NEJMoa1605215 27602507

[73] Janjua T Narvaez AR Florez-Perdomo WA Guevara-Moriones N Moscote-Salazar LR. A review on decompressive craniectomy for traumatic brain injury: the mainstay method for neurotrauma patients. Egypt J Neurosurg. (2023) 38:75. doi: 10.1186/s41984-023-00237-6

[74] Mohan M Layard Horsfall H Solla DJF Robertson FC Adeleye AO Teklemariam TL. Decompressive craniotomy: an international survey of practice. Acta Neurochir. (2021) 163:1415–22. doi: 10.1007/s00701-021-04783-6, PMID: 33738561 PMC8053664

[75] Hawryluk G Rubiano AM Totten AM O’Reilly C Ullman JS Bratton SL. Update of the Decompressive Craniectomy recommendations. Neurosurgery. (2020) 87:427–34. doi: 10.1093/neuros/nyaa278, PMID: 32761068 PMC7426189

[76] Menon DK Ercole A. Critical care management of traumatic brain injury. Handb Clin Neurol. (2017) 140:239–74. doi: 10.1016/B978-0-444-63600-3.00014-3 28187802

[77] Negida A Teton Z Stedelin B Nerison C Al-Shami H Hegazy A. Determining the global outcomes of traumatic brain injury in low-, middle-and high- income countries: a prospective, global neurosurgery, multicenter cohort study (global neuro Surg 1 study protocol). J Surg Protoc Res Methodol. (2021) 2021:snab002. doi: 10.1093/jsprm/snab002

[78] Fatima N Al Rumaihi G Shuaib A Saqqur M. The role of decompressive craniectomy in traumatic brain injury: a systematic review and meta-analysis. Asian J Neurosurg. (2019) 14:371–81. doi: 10.4103/ajns.AJNS_289_18, PMID: 31143249 PMC6515989

[79] Cucciolini G Motroni V Czosnyka M. Intracranial pressure for clinicians: it is not just a number. J Anesth Analg Crit Care. (2023) 3:31. doi: 10.1186/s44158-023-00115-5, PMID: 37670387 PMC10481563

[80] Patel S Maria-Rios J Parikh A Okorie ON. Diagnosis and management of elevated intracranial pressure in the emergency department. Int J Emerg Med. (2023) 16:72. doi: 10.1186/s12245-023-00540-x, PMID: 37833652 PMC10571389

[81] Petersson L Larsson I Nygren JM Nilsen P Neher M Reed JE. Challenges to implementing artificial intelligence in healthcare: a qualitative interview study with healthcare leaders in Sweden. BMC Health Serv Res. (2022) 22:850. doi: 10.1186/s12913-022-08215-8, PMID: 35778736 PMC9250210

[82] Nag DS Sahu S Swain A Kant S. Intracranial pressure monitoring: gold standard and recent innovations. World J Clin Cases. (2019) 7:1535–53. doi: 10.12998/wjcc.v7.i13.1535, PMID: 31367614 PMC6658373

[83] Stein SC Burnett MG Glick HA. Indications for CT scanning in mild traumatic brain injury: a cost-effectiveness study. J Trauma. (2006) 61:558–66. doi: 10.1097/01.ta.0000233766.60315.5e, PMID: 16966987

[84] Waterhouse C. Practical aspects of performing Glasgow coma scale observations. Nurs Stand. (2017) 31:40–6. doi: 10.7748/ns.2017.e10189, PMID: 28443443

[85] Robba C Graziano F Rebora P Elli F Giussani C Oddo M. Intracranial pressure monitoring in patients with acute brain injury in the intensive care unit (SYNAPSE-ICU): an international, prospective observational cohort study. Lancet Neurol. (2021) 20:548–58. doi: 10.1016/S1474-4422(21)00138-1 34146513

[86] Lele A Kannan N Vavilala MS Sharma D Mossa-Basha M Agyem K. Patients who benefit from intracranial pressure monitoring without cerebrospinal fluid drainage after severe traumatic brain injury. Neurosurgery. (2019) 85:231–9. doi: 10.1093/neuros/nyy247, PMID: 30053135

[87] Yuan Q Wu X Cheng H Yang C Wang Y Wang E. Is intracranial pressure monitoring of patients with diffuse traumatic brain injury valuable? An observational multicenter study. Neurosurgery. (2016) 78:361–368; discussion 368-9. doi: 10.1227/NEU.0000000000001050 26891376

[88] Yuan Q Wu X Yu J Sun Y Li Z Du Z. Effects and clinical characteristics of intracranial pressure monitoring-targeted management for subsets of traumatic brain injury: an observational multicenter study. Crit Care Med. (2015) 43:1405–14. doi: 10.1097/CCM.0000000000000965, PMID: 25803654

[89] Bennett TD Riva-Cambrin J Keenan HT Korgenski EK Bratton SL. Variation in intracranial pressure monitoring and outcomes in pediatric traumatic brain injury. Arch Pediatr Adolesc Med. (2012) 166:641–7. doi: 10.1001/archpediatrics.2012.322, PMID: 22751878 PMC4547349

[90] Mauritz W Steltzer H Bauer P Dolanski-Aghamanoukjan L Metnitz P. Monitoring of intracranial pressure in patients with severe traumatic brain injury: an Austrian prospective multicenter study. Intensive Care Med. (2008) 34:1208–15. doi: 10.1007/s00134-008-1079-7, PMID: 18365169

[91] Lane PL Skoretz TG Doig G Girotti MJ. Intracranial pressure monitoring and outcomes after traumatic brain injury. Can J Surg. (2000) 43:442–8. https://www.ncbi.nlm.nih.gov/pmc/articles/PMC3695200 11129833 PMC3695200

